# Variability in DNA COI sequences reveals new haplotypes in freshwater turtles from northern region of India

**DOI:** 10.1080/23802359.2018.1443850

**Published:** 2018-03-07

**Authors:** Ranjana Bhaskar, Vindhya Mohindra

**Affiliations:** ICAR-National Bureau of Fish Genetic Resources, Lucknow, Uttar Pradesh, India

**Keywords:** Cytochrome c oxidase I, divergence, haplotype, clades

## Abstract

The chelonian represents a diverse order of turtles. Little is known about this group from India at molecular level. Cytochrome c oxidase I (COI) sequences of 125 individuals from nine species of freshwater turtles were generated and analyzed. A total of 118 nucleotide positions in *COI* gene as character-based DNA barcodes for each species were identified. Neighbour-joining (NJ) analyses tree well differentiated freshwater hard shell and softshell turtle. Overall species divergence (K2P) was 13.3%. Analysis of COI sequences from present study, combined with sequences downloaded from NCBI GenBank, revealed new COI haplotypes from Northern region of India.

## Introduction

India has a rich diversity of turtles, which belong to the order Chelonia, with 13 recognized families in the world and out of these five families present in India. A total of 29 species of freshwater turtles and tortoises are found in India (Ramakrishna et al. 2014), 15 of which have been reported from the northern states of India, and Uttar Pradesh (U.P.). Illegal trade of these turtles is prevalent in India , and due to its high demand in the international market. However, the baseline information of the species distribution patterns is limited (Singh et al. 2009). Due to their commercial exploitation, many freshwater species inhabiting India are listed in Convention on International Trade in Endangered Species (CITES) and Wildlife (Protection) Act 1972.

The present study aimed at identification of turtle species across the geographical range in U.P. state, India and analysis of genetic variation present in different species using cytochrome c oxidase I (*COI*) gene sequences. Out of nine species studied, five species are Endangered and protected under the Wildlife (Protection) Act 1972, in schedule I. Downloaded sequences from NCBI, from a previous study, were also added in this study to evaluate variability and identification success across the geographical range of Uttar Pradesh. The current study also concludes the existence of few species in the wild, although they are already declared as Endangered in IUCN (International Union for Conservation of Nature).

## Methodology

**Figure 1.**
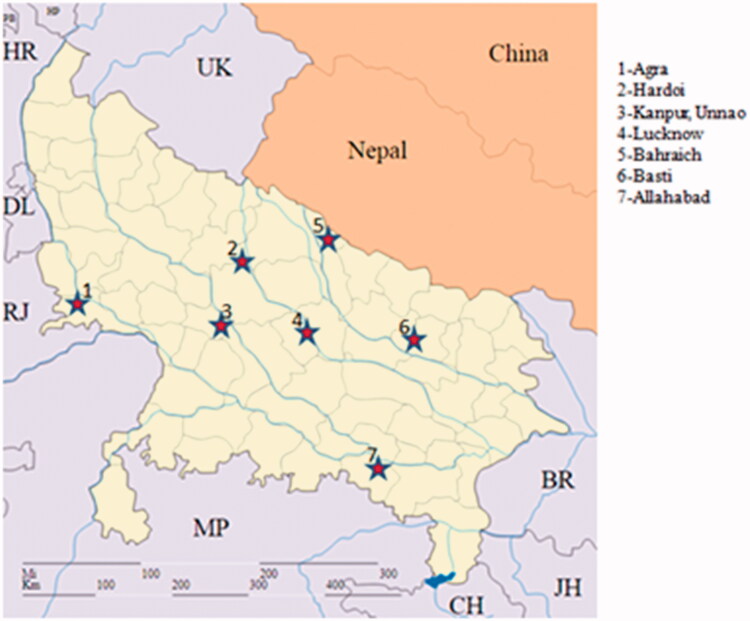
Sample collection sites in Uttar Pradesh, India (filled star) for freshwater turtle.

Samples were collected from 125 individuals (non-invasive) of nine turtle species in this study, which belong to two families: Trionychidae and Geoemydidae, from natural locations of Uttar Pradesh, India (Figure 1, Table 1). The procedure followed was approved by Institute's Animal Ethics Committee. Genomic DNA was extracted using standard phenol-chloroform extraction method. Approximately 100 ng of DNA was used for the PCR amplification using primers FishF1 (TCAACCAACCACAAAGACATTGGCAC) and FishR1 (TAGACTTCTGGGTGGCCAAAGAATCA) (Ward et al. 2005) for all species. Polymerase chain reaction (PCR) was performed in (ABI Thermo Cycler) a final volume of 25 µl reaction containing 1× PCR Buffer, 5 mM MgCl_2_; 10 mM dNTPs; 5 pmol of each primer (Sigma Genosys Ltd.); 1U Taq polymerase (Merk, India). Amplification conditions were 94°C for 5 min, followed by 30 cycles at 94°C for 1 min, annealing at 55°C (Ta) for 1min and 72°C for 1min, with final extension of 72°C for 10 min and stored at 4°C. PCR products were sequenced bi-directionally, using an automated capillary sequencer (ABI377).

**Table 1.** Species, sample size, and location of collection site of freshwater turtle from different rivers in Uttar Pradesh and sequences submitted and downloaded from NCBI.

Species	River	Collection site	Location (lat. & log.)	GenBank accession Numbers, COI
*L. punctata*	Kuwano	Basti (5)	26° 81′N, 82° 76′E	MF432727-MF432785
	Indra nahar	Nigoha Lucknow (3)	26° 56′N, 81° 02′E	
	Gomti	Hardoi (3)	27° 39′N, 80° 13′E	
	Gomti	Lucknow city (7)	26° 84′N, 80° 94′E	
	Ganga	Kanpur (6)	26° 44′N, 80° 33′E	
	Sai Nadi	Maurawa, Unnao (15)	26° 53′N, 80° 48′E	
	Yamuna river	Agra (3)	27° 17′N, 78° 00′E	
	Ganga	Allahabad (5)	25° 43′N, 81° 84′E	
	Sarju	Bahraich (1)	27° 86′N, 81° 49′E	
	Pond	Bachrawa (7)	26° 47′N, 81° 11′E	
*N. Gangaticus*	Ganga	Allahabad (6)	25° 43′N, 81° 84′E	MF432821-MF432839
	Sarju	Bahraich (1)	27° 86′N, 81° 49′E	
	Yamuna	Agra (12)	27° 17′N, 78° 00′E	
*N. hurum*	Kuwano	Basti (1)	26° 81′N, 82° 76′E	MF432840, MF432841
	Yamuna	Agra (2)	27° 17′N, 78° 00′E	
*C. indica*	Ganga	Allahabad (6)	25° 43′N, 81° 84′E	MF432789-MF432797
	Yamuna	Agra (4)	27° 17′N, 78° 00′E	
*K. smithii*	Ganga	Phaphamau Allahabad (7)	25° 53′N, 81° 87′E	MF432800-MF432806
	Sai nadi	Maurawa, Unnao (2)	26° 06′N, 81° 44′E	
*H. thurjii*	Sai nadi	Maurawa, Unnao (3)	26° 06′N, 81° 44′E	MF432798, MF432799
*K. tecta*	Sai nadi	Maurawa, Unnao (3)	26° 06′N, 81° 44′E	MF432807-MF432815
	Kuwano	Basti (1)	26° 81′N, 82° 76′E	
	Sarju	Bahraich (4)	27° 86′N, 81° 49′E	
*K. tentoria*	Sai Nadi	Maurawa, Unnao (2)	26° 06′N, 81° 44′E	MF432816-MF432820
	Gomti	Hardoi (3)	27° 39′N, 80° 13′E	
*B. kachuga*	Kukrail picnic spot, Lucknow	Kukrail, Lucknow (1)	26° 90′N, 80° 98′E	MF432786-MF432788
	Gomti	Hardoi (2)	27° 39′N, 80° 13′E	
*L. punctata*		Assam		KF894768-69, KF89476,
		American Museum of Natural History		HQ329775
		West Bangal		JN794085-88, JX049143
		Tripura		JN416995-98
*N. gangaticus*		American Museum of Natural History		HQ329780
		Assam		JN416999
		Tripura		JN416993 KC311378-79
*N. hurum*		Tripura		JN416996, KC311383 KC311380
		American Museum of Natural History		HQ329781
		Assam		GU563918
*C. indica*		Assam		JN416992 KF894787 KC311376-77
		American Museum of Natural History		HQ329771
*K. smithii*		American Museum of Natural History		HQ329694
		Assam		KF894780-81
*K. tentoria*		Assam		JN860214-16
		American Museum of Natural History		HQ329696
		Assam		KF894784-86
*K. tecta*		Meghalaya		JN680211
		American Museum of Natural History		HQ329695
		Assam		KF894782-83
*H. thurjii*		Assam		KF894763-64
		American Museum of Natural History		HQ329680
*B. kachuga*		Assam		KF894755-56
		American Museum of Natural History		HQ329674

Individual consensus sequences were edited by BioEdit and ClustalW (http://www.clustaLissemysorg/clustal2/) and all sequences were aligned with numts DNA sequences to identify numts. Mitochondrial haplotype (h) and nucleotide (p) diversities were calculated using the software DNASP (v3.0; Rozas et al. 2003). All statistical parameters were calculated using the program MEGA 6.0 (Tamura et al. 2007). The sequences for other species (*Lissemys punctata, Nilssonia gangetica, Chitra indica, N. hurum, Kachuga tentoria, K. tecta, K. smithii, Hardella thurjii thurjii*, *and Batagur kachuga*) were downloaded from GenBank and were used in the analysis (Table 1).

## Results

A total of 115 COI sequences were generated from nine species were of freshwater turtles, *L. punctata, N. gangetica, C. indica, N. hurum, K. tentoria, K. tecta, K. smithii, H. thurjii thurjii*, *and B. kachuga* (Table 1). All are submitted in (680 bp, Accession No. MF432727-MF432841) NCBI GenBank.

Out of the 680 bp analyzed, 453 bp (66.61%) conserved, 227 (33.38%) variable, 6 (0.88%) singleton, and 221 (32.5%) were parsimony informative. The average nucleotide base composition was 28.5% T, 26.4% C, 29.0% A, and 16.2% G, with a total T and A contents (57.5%) higher than that of C and G (42.6%). Twenty four mtDNA haplotypes were identified and variable sites of COI fragments are shown in Table 2. The overall haplotype diversity was 0.723 and nucleotide diversity was 0.1151 (Table 3). The numbers of haplotypes within the species are shown in Table 3.

**Table 2.** The haplotype diversity and pure diagnostic characters at selected nucleotide positions of COI fragments in nine species of freshwater turtle.

**Table 3.** Number of haplotypes, haplotype diversity (h), and nucleotide diversity (p), with sample size of COI for the freshwater turtle.

Species	Haplotypes	Haplotype diversity	Nucleotide diversity	Sample size
*K. tecta*	3	0.6430 ± 0.1840	0.00150 ± 0.00058	8
*H. thurjii*	1	0.0000 ± 0.0000	0.00000 ± 0.00000	3
*K. smithii*	3	0.3330 ± 0.2150	0.00050 ± 0.00032	7
*K. tentoria*	3	0.7000 ± 0.2180	0.00150 ± 0.00052	5
*N. hurum*	1	0.0000 ± 0.0000	0.00000 ± 0.00000	3
*C. indica*	4	0.0000 ± 0.0000	0.00000 ± 0.00000	9
*N. ganegetica*	3	0.2050 ± 0.2050	0.00063 ± 0.00043	19
*L. punctata*	5	0.1620 ± 0.0640	0.00025 ± 0.00010	59
*B. kachuga*	1	0.0000 ± 0.0000	0.00000 ± 0.00000	2

Character-based DNA barcodes (COI) were identified using unique combinations of character at 118 nucleotide positions (Table 2) (Naro-Macil et al. 2010). *Lissemys punctata* was separated with highest pure diagnostic characters of 43, while three, nine, and eleven characters (CAs) defined in *K. tentoria*, *K. smithii*, and *C. indica*. Fifteen and 12 diagnostic characters were found in *N. gangaticus* and *N. hurum*, respectively. *Nilssonia gangaticus* was diagnosed by C and T at positions 269 and 284, while *N. hurum* by T and C at the same positions. At positions 623, C, T, and G were identified in *N. gangaticus, N. hurum, and K. smithii*. The interspecies transition (ti) and transversion (tv) value was 602 and 44, respectively, while overall ti/tv ratio was 1.418. Pairwise Kimura 2-parameter (K2P) sequence divergence comparison between the nine species were 0.037 (*K. tentoria* and *K. smithii*) to 0.313 (*K. tecta* and *L. punctata*) (Table 4). Species *K. tecta* and *L. punctata* had highest genetic distance (0.313) with each other.

**Table 4.** Divergence values for COI between the freshwater turtle species.

	*B.kachuga*	*K. tecta*	*H. thurjii*	*K. smithii*	*K. tentoria*	*N. hurum*	*C. indica*	*N. ganegetica*	*L. punctata*
*B. kachuga*	*0.001*	0.115	0.081	0.101	0.109	0.171	0.144	0.168	0.191
*K. tecta*	0.139	*0.001*	0.127	0.073	0.072	0.186	0.163	0.179	0.199
*H. thurjii*	0.093	0.157	*0.000*	0.114	0.117	0.165	0.149	0.164	0.187
*K. smithii*	0.119	0.083	0.139	*0.001*	0.035	0.183	0.158	0.179	0.196
*K. tentoria*	0.129	0.081	0.142	0.037	*0.001*	0.185	0.156	0.184	0.192
*N. hurum*	0.246	0.277	0.234	0.270	0.278	*0.000*	0.119	0.081	0.166
*C. indica*	0.193	0.228	0.169	0.218	0.213	0.149	*0.002*	0.125	0.159
*N. ganegetica*	0.242	0.262	0.200	0.264	0.273	0.091	0.157	*0.001*	0.172
*L. punctata*	0.294	0.313	0.279	0.306	0.298	0.238	0.218	0.246	*0.001*
*G. gangeticus*	0.230	0.249	0.254	0.242	0.237	0.230	0.234	0.242	0.253

Average within-species divergence calculated using the Kimura 2-parameter model (K2P) is on the diagonal and given in italics. Average pair wise divergences between species p-distance above the diagonal, K2P divergence: below diagonal.

**Figure 2.**
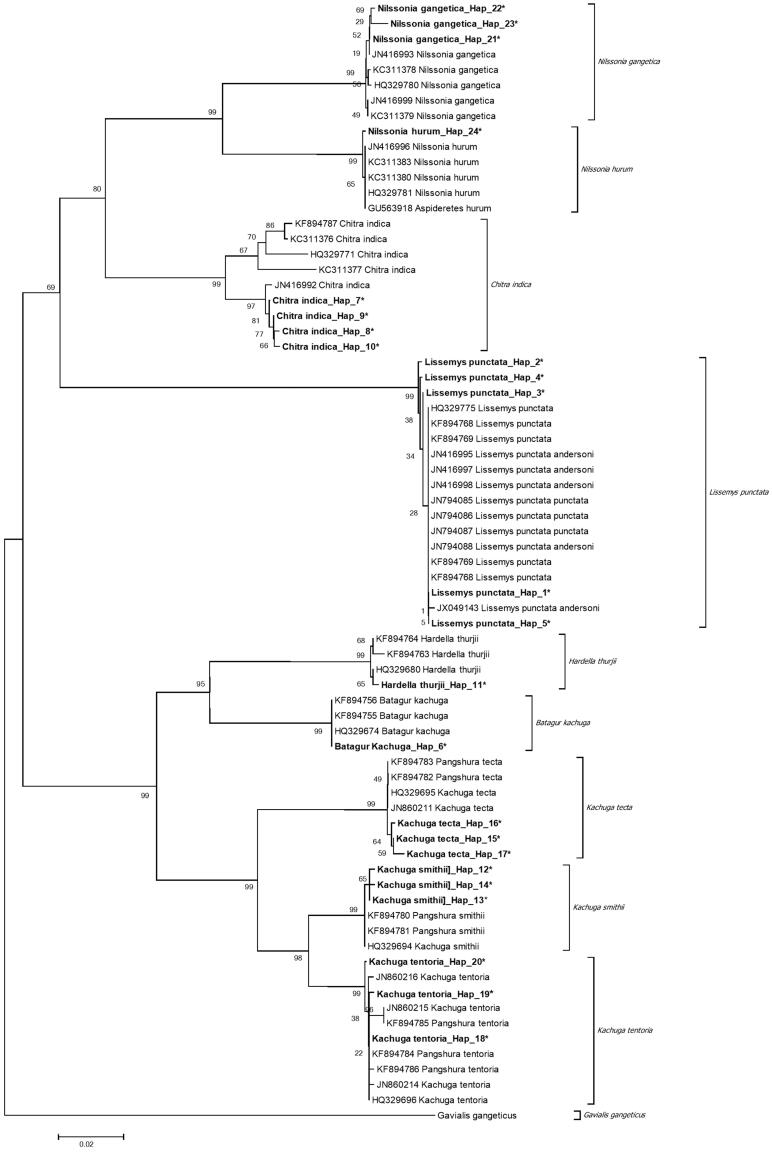
Sequences generated in this study (*) and downloaded COI sequences from NCBI showed the differentiation from NCBI data and indicate the new haplotypes for present study (*). The evolutionary distances were computed using the Kimura 2-parameter method and are in the units of the number of base substitutions per site. Numbers near corresponding branches indicate NJ percentages out of 1000 bootstrap replicates.

The overall species (K2P) diversity 13.3%. NJ was observed three major clusters in nine species of freshwater turtles and out group with high bootstrap value (Figure 2). The first cluster contained softshell turtle, second hardshell turtle, and third out group. The first cluster contained softshell turtle belonging to genus *Nillsonia, Chitra,* and *Lissemys* (*L. punctata, N. gangetica, C. indica, N. hurum*) with 100% bootstrap support (100% in ML and 74% in NJ) and another cluster contained genus *Kachuga* (*K. tentoria, K. tecta*, and *K. smithii*)*, Hardella* (*H thurjii thurjii*), and *Batagur* (*B. kachuga*) with high bootstrap value.

Analysis of sequences generated in present study and 48 sequences downloaded from NCBI revealed NJ tree with high bootstrap value (Figure 2), indicating the presence of a new haplotype for North Indian species.

## Discussion

This study of nine species of Indian freshwater turtles included two families, Trionychidae (softshell) and Geoemydidae (hardshell). It was observed that character-based diagnosis generated was reliable for the identification of closely related species. The nucleotide compositions revealed a bias against G and transitions were more frequent than transversions. This bias has been reported to be widespread in turtle as compared to animal mitochondrial DNA and follows very different patterns of evolution (Belle et al. 2005).

The well-supported trees for the phylogenetic relationships differentiated all the species. New COI North-India haplotypes were observed in the present study, when compared to previous sequences from NCBI, which are mostly from Assam State, Northeast India (Kundu et al. 2016). Five out of nine species in this study were reported as Endangered in scheduled I in (Protection) Act 1972 and all of these are commercially exploited. Present study clearly showed the new haplotypes in all the species except two haplotypes in *L. punctata* (Hap 1,5) and *K. tentoria* (Hap 18,19) and one in *B. kachuga* (Hap 6) (Figure 2), indicating the North Indian population that are well differentiated from Northeast Indian population and this can form basic information for North Indian freshwater species and has forensic applications, as well as useful for identification, management of natural populations, and also resolving taxonomic ambiguity. This is the first time study on North Indian freshwater turtles.
